# Comparison of DNA sequencing and morphological identification techniques to characterize environmental fungal communities

**DOI:** 10.1038/s41598-021-81996-w

**Published:** 2021-01-29

**Authors:** Naohide Shinohara, Cheolwoon Woo, Naomichi Yamamoto, Kazuhiro Hashimoto, Hiroko Yoshida-Ohuchi, Yuji Kawakami

**Affiliations:** 1 Research Institute of Science for Safety and Sustainability (RISS), National Institute of Advanced Industrial Science and Technology (AIST), 16-1 Onogawa, Tsukuba, Ibaraki 305-8569 Japan; 2 Department of Environmental Health Sciences, Graduate School of Public Health, Seoul National University, Seoul, 08826 Republic of Korea; 3 Laboratory of Integrated Pest Management, FCG Research Institute Inc., 1-1-20Koto-ku, Aomi, 135-0064 Japan; 4 Graduate School of Pharmaceutical Sciences, Tohoku University, 6-3 Aramaki-Aoba, Aoba-ku, Sendai, Miyagi 980-8578 Japan

**Keywords:** Microbiology, Environmental sciences

## Abstract

Culture-independent DNA sequencing of fungal internal transcribed spacer 2 (ITS2) region was compared to a culture-dependent morphological identification technique to characterize house dust-borne fungal communities. The abundant genera were *Aspergillus*, *Wallemia*, *Cladosporium*, and *Penicillium*. Statistically significant between-method correlations were observed for *Wallemia* and *Cladosporium* (Spearman’s *ρ* = 0.75 and 0.72, respectively; *p* < 0.001). *Penicillium* tended to be detected with much higher (averaged 26-times) relative abundances by the culture-based method than by the DNA-based method, although statistically significant inter-method correlation was observed with Spearman’s *ρ* = 0.61 (*p* = 0.002). Large DNA sequencing-based relative abundances observed for *Alternaria* and *Aureobasidium* were likely due to multicellularity of their spores with large number of per-spore ITS2 copies. The failure of the culture-based method in detectiing *Toxicocladosporium*, *Verrucocladosporium*, and *Sterigmatomyces* was likely due to their fastidiousness growth on our nutrient medium. Comparing between the two different techniques clarified the causes of biases in identifying environmental fungal communities, which should be amended and/or taken into consideration when the methods are used for future fungal ecological studies.

## Introduction

Fungi are ubiquitous in the indoor environment^1^. Indoor dampness can cause fungal infestation that might be linked to respiratory illnesses, such as asthma^2–4^, rhinitis^3,5^, and upper respiratory tract symptoms^2^. Accurate identification of fungi in indoor environments may therefore be critical. To assess fungal exposure and associated health outcomes, fungal levels in indoor air or settled dust are often determined^1^. Fungal communities in the air temporally fluctuate, whereas those in settled dust represent the time-integrated communities that are temporally more stable^1^. Among numerous methods available for settled dust sampling, such as swab^6^ and wipe^7^, vacuuming can collect large quantities of dust samples from entire residential spaces regardless of flooring types (e.g., carpeted and hard floors).

Traditionally, culture-based approaches have been used to analyze microorganisms in indoor environments, including settled floor dust samples^1^. However, this approach can be biased, for example, by microbial viability and/or culturability on a given nutrient medium^1^. The advent of growth-independent molecular biology-based techniques, such as polymerase chain reaction (PCR) and DNA sequencing, has circumvented these difficulties^1^. However, few studies have directly compared culture-based morphological identification methods with culture-independent DNA sequencing-based approaches. For example, a previous study compared the presence or absence of fungal species detected by a culture-based morphological identification method and a culture-independent DNA sequencing method^8^; however, only a qualitative comparison was conducted between these two different approaches and a quantitative comparison was not conducted.

In the present study, we aimed to compare a culture-dependent morphological identification method and a culture-independent DNA sequencing method to quantify fungal communities detected from settled floor dust samples, which were previously collected using a vacuuming method from a total of 24 homes close to the Fukushima Daiichi Nuclear Power Plant in Fukushima Prefecture, Japan^9,10^. The collected settled dust samples were analyzed via a conventional culture-based macroscopic plus microscopic morphological identification method, as well as high-throughput amplicon sequencing of fungal internal transcribed spacer 2 (ITS2) region^11^, as a DNA metabarcoding maker for the Fungi kingdom^12^ on an Illumina MiSeq platform.

## Results

### Culture-based results

Fungal concentrations in house dust varied widely among houses (mean ± SD: 2.1 × 10^7^ ± 1.7 × 10^7^ CFU/g-dust; median: 1.5 × 10^7^ CFU/g-dust). In most houses, *Aspergillus* (prevalence 100%), *Penicillium* (prevalence 92%), *Cladosporium* (prevalence 92%), and *Wallemia* (prevalence 79%) were observed (Supplementary Table S1). The mean relative abundances of the fungal genera *Aspergillus*, *Wallemia*, *Penicillium*, and *Cladosporium* were 51%, 8.1%, 7.3%, and 4.1%, respectively (Supplementary Table S1). Approximately 30% of fungal species could not be identified.

Among *Aspergillus* species, *A. penicillioides*, *A. vitricola*, *A. restrictus*, *A.* section *Restricti—*i.e., a type of species other than the previously listed species—, *A. ochraceus*, *A. sclerotiorum*, *A. versicolor*, and *A. sydowii* were often detected (Supplementary Fig. S1). Among *Wallemia* species, only *W. sebi* was detected.

### DNA sequencing-based results

According to the UNITE (and RefSeq) databases, 1,556 (and 1,316) fungal genera were detected in more than one house, and 46 (49), including *Aspergillus*, *Wallemia*, *Cladosporium*, *Verrucocladosporium*, *Sterigmatomyces*, and *Toxicocladosporium*, were detected in all 24 houses (prevalence 100%).

Additionally, according to the UNITE (RefSeq) databases, the mean relative abundances of the fungal genera *Aspergillus*, *Wallemia*, *Cladosporium*, *Verrucocladosporium*, *Rasamsonia*, *Sterigmatomyces*, *Geosmithia*, *Toxicocladosporium*, and *Chrysosporium* were 33% (35%), 13% (13%), 8.8% (8.8%), 2.9% (2.9%), 2.9% (2.4%), 2.4% (2.4%), 2.3% (2.3%), 1.8% (1.8%), and 1.7% (1.8%), respectively (Supplementary Table S1). The relative abundance of most genera assessed based on the analyses of both databases were well correlated, with a slope of 1. However, no correlation was found between different genera, including *Pseudopithomyces*, *Epicoccum*, *Exobasidium*, and *Lecanicillium*, (Supplementary Fig. S2).

### Relationships between culture- and DNA sequencing-based results

**Figure 1:**
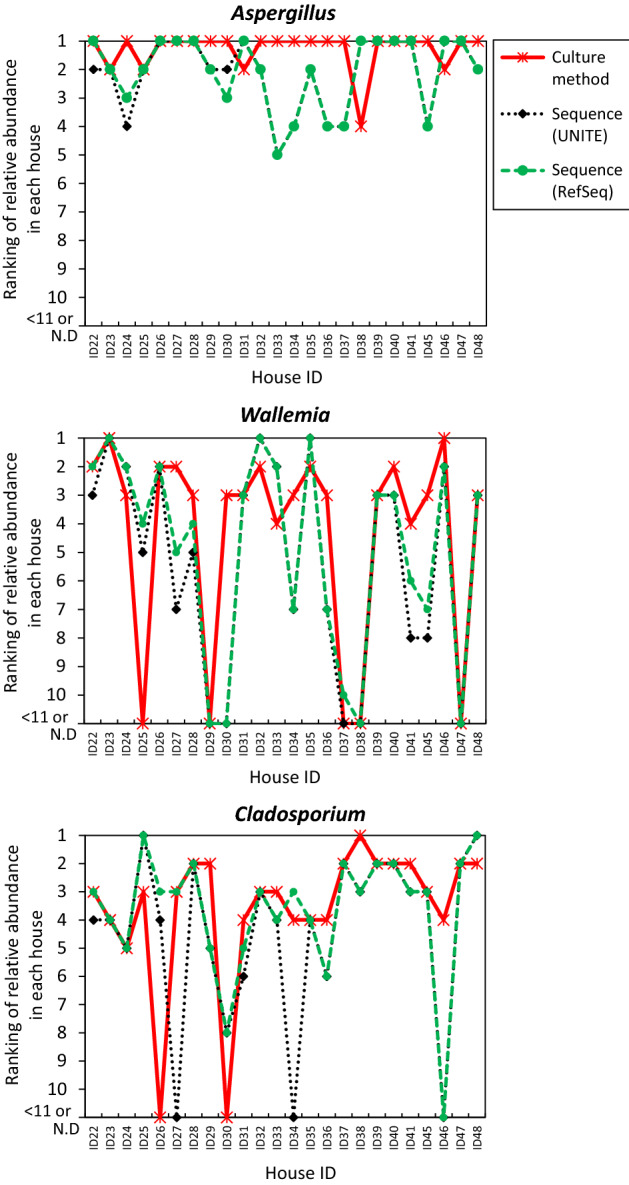
Rankings of the relative abundances in each house. (Upper) *Aspergillus*, (Middle) *Wallemia*, (Lower) *Cladosporium*.

**Figure 2:**
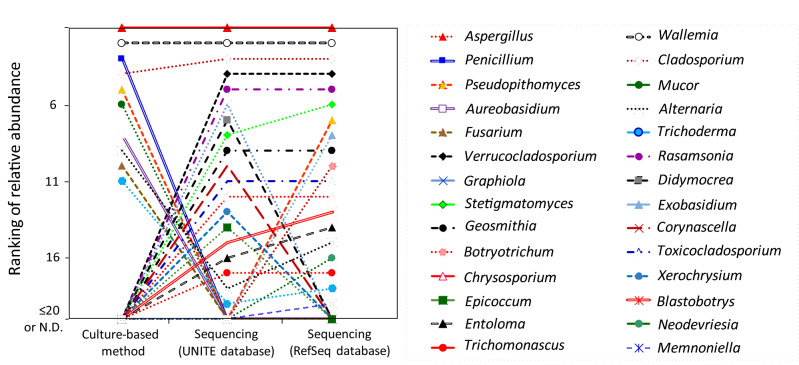
Rankings of averaged relative abundances of major fungal genus.

Ranking the average relative abundances, we found that the genera *Aspergillus*, *Wallemia*, and *Cladosporium* were 1st, 2nd, and 4th with culture and 1st, 2nd, and 3rd with sequencing, respectively (Fig. 1 and Supplementary Table S1). In each house, the abundance ratio rankings were similar between the culture and sequencing for *Aspergillus*, *Wallemia*, and *Cladosporium* (Fig. 2).

**Figure 3:**
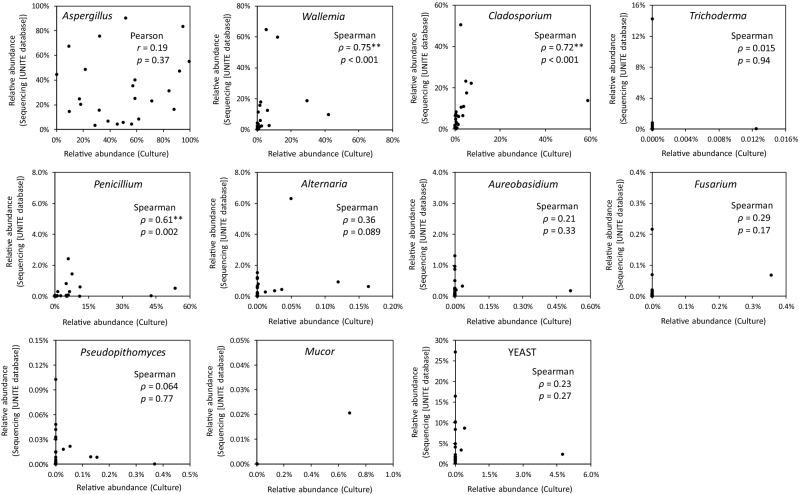
Quantitative comparison of methods. For each fungal genus, the correlation of the relative abundances detected by the culture and sequencing methods is shown.

An overview of relative abundances detected by the sequencing and culture methods, and the results of analyses of principal components, are shown in Fig. 3. *Aspergillus*, *Wallemia*, *Cladosporium*, and *Penicillium* were detected both through the DNA sequencing (against the UNITE database) and culture methods at high relative abundances, although the relative abundances of *Penicillium* through DNA sequencing were much lower than those in culture. *Alternaria* and *Aureobasidium* were mostly detected through DNA sequencing, whereas these showed quite low relative abundance or were not detected by the culture method. *Verrucocladosporium*, *Rasamsonia*, *Graphiola*, *Didymocrea*, *Sterigmatomyces*, and *Toxicocladosporium*, were detected in most of houses through DNA sequencing, but these were not detected in the culture. *Pseudopithomyces* showed inconsistent results between both methods although this fungus was detected using both methods.

For all fungal genera, except for *Aspergillus*, both methods yielded non-normally distributed relative abundances. For *Aspergillus*, no significant Pearson correlation was found between the relative abundances determined by the two methods (Pearson *r* = 0.19; *p* = 0.37; Fig. 3). On average, the estimated relative abundance of *Aspergillus* was significantly higher with the culture method than with DNA sequencing (paired *t*-test; *p* = 0.021).

Spearman’s rank correlation showed significance for *Wallemia*, *Cladosporium*, and *Penicillium* (*ρ* = 0.75; *p* < 0.001, *ρ* = 0.72; *p* < 0.001, and *ρ* = 0.61; *p* = 0.002, respectively). *Wallemia* was found to be more abundant after DNA sequencing than with the culture method, though the difference was not significant (Wilcoxon signed-rank test *p* = 0.072). *Cladosporium* was significantly more abundant with DNA sequencing than with culture (Wilcoxon signed-rank test *p* < 0.001). For *Penicillium*, the estimated relative abundance was clearly lower with sequencing than with culture (Wilcoxon signed-rank test *p* < 0.001). Although 61 genera of yeasts, including *Sterigmatomyces*, *Blastobotrys*, *Trichomonascus*, *Debaryomyces*, *Candida*, *Yamadazyma*, and *Cryptococcus*^13,14^, were detected by DNA sequencing in the present study, the sum of the relative abundances of these genera was higher after sequencing compared to with the culture (Wilcoxon signed-rank test; *p* < 0.001), and the two methods showed no significant correlation (Spearman’s *ρ* = 0.23; *p* = 0.27).

## Discussion

Herein, we compared the performance of the culture- and DNA sequencing-based techniques to characterize environmental fungal communities. The culture method has an apparent limitation in the number of analyzable colonies—i.e., 100–200 colonies per plate—resulting in lower limits of detection of 0.5%–1% relative abundances. Other limitations include its inability to detect non-viable spores or cells^1^, difficulty culturing fastidious species on a given nutrient medium^1^, and difficulty distinguishing sibling taxa that have similar morphological characteristics^15,16^. Meanwhile, the DNA sequencing suffers from a bias associated with copy number variation in the target DNA marker (ITS2) across species and strains^17,18^. Another caveat lies in the uncertainty of the accuracy of curation and taxonomic coverage of reference databases.

**Figure 4:**
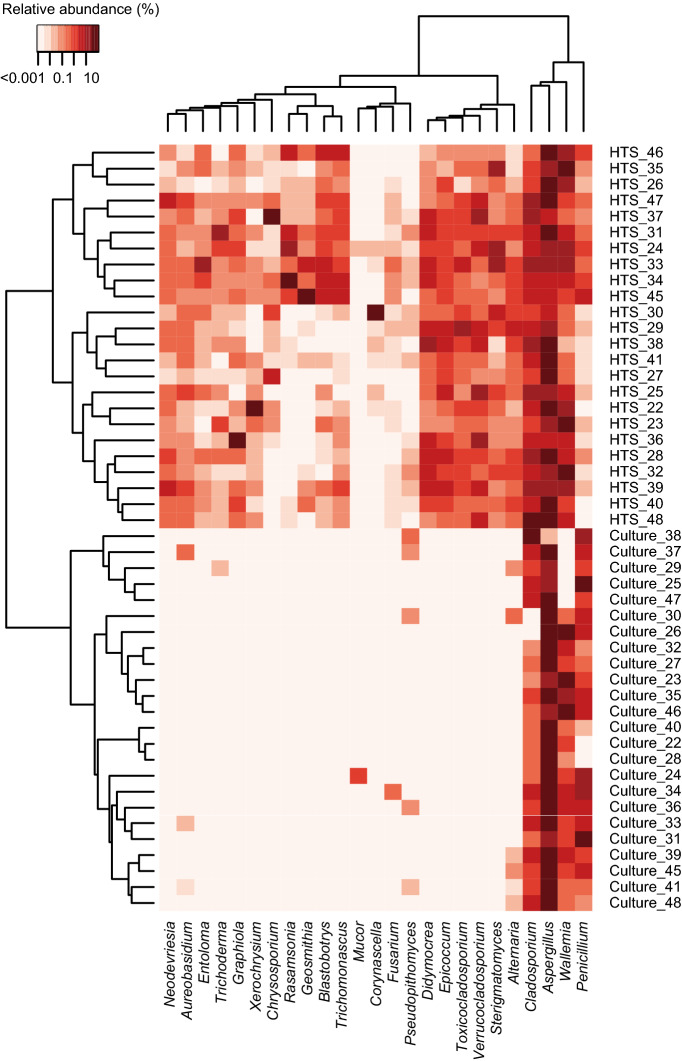
Relative abundances of selected fungal genera. The top 20 most abundant genera detected by DNA sequencing against the UNITE database (genera detected by culture method are also included). The trees represent Euclidean distances based on log-transformed relative abundances.

Statistically significant inter-method correlations were observed for *Wallemia* and *Cladosporium* with Spearman’s *ρ* = 0.75 and 0.72 (*p* < 0.001), respectively. However, the DNA-based relative abundances were 1.6- and 2.2-fold higher than the culture-based relative abundances of *Cladosporium* and *Wallemia*, respectively, indicating that the culture-based method underestimated their relative abundances. One possible cause is selective loss in their viability. For instance, a study reported lower viability of *Cladosporium* than those of *Aspergillus* and *Penicillium*^19^, whereas another study reported rapid loss of viability of *Wallemia sebi* as compared to *Aspergillus* spp.^20^. The selective loss of viability might be a cause of the underestimation of these genera by the culture-based approach. The culture-based method also underestimated relative abundances of *Alternaria* and *Aureobasidium*. Similarly, *Epicoccum* was detected from all samples thorough the DNA-based method, but was undetected using the culture-based method (Fig. 4). These fungi are known to form large multicellular spores^21,22^. Due to the multicellularity of their spores with large number of per-spore ITS2 copies, their colony forming units were likely underestimated as compared to estimation of the number of ITS2 copies using the DNA-based approach.

The culture-based method failed to detect several fungal genera that were detected by the DNA-based method (Fig. 4). We expect that this failure was partly due to their fastidiousness of growth on our nutrient medium (DG18). For instance, the culture-based technique failed to detect several ascomycete genera such as *Verrucocladosporium* and *Toxicocladosporium* that are known to be slow-growing even on water-rich media including potato dextrose agar (PDA) and malt extract agar (MEA)^23,24^. The culture-based technique also failed to detect several basidiomycete genera, including *Entoloma* that is known to grow well only on water-rich media such as Murashige and Skoog medium and PDA^25,26^. Thus, the DG18 medium used in this study is also likely unsuitable for other basidiomycetes such as *Graphiola*, *Sterigmatomyces*, and *Exobasidium* that were detected only using the DNA-based technique.

No significant correlation was observed for relative abundances of the genus *Aspergillus* between the culture- and DNA-based techniques. One possible cause is the inter-species variability in preference of our nutrient medium^27^ since *Aspergillus* contains xerophilic species (e.g., *Aspergillus halophilicus*) that are known to show poor growth on DG18^16^. Another possible cause is inter-sample variability in aspergilli viability, which could disproportionately affect culture-based detection. These inter-species variability in their culturability and inter-sample variability in their viability might obscure the correlation in their abundances based on the culture- and DNA-based techniques.

*Penicillium* tended to be detected with much higher (26-fold) relative abundances on average thorough the culture-based method than the DNA-based method; however, this statistically significant inter-method correlation was observed with Spearman’s *ρ* = 0.61 (*p* = 0.002) (Fig. 3). The discrepancy might be in part due to the misidentification by morphological observation, for example, with *Geosmithia*, a *Penicillium*-like genus^15^. Additionally, the discrepancy is likely attributable to the database bias of DNA-based identification. If the previous version of UNITE database (reference database UNITEdatabaseinFHiTINGSformat20-11-2016release.fasta) was used, then no statistical correlation was observed between the two methods (Spearman’s *ρ* = 0.23; *p* = 0.27; Supplementary Fig. S3). We observed no correlation between the previous version of the UNITE and RefSeq databases (Supplementary Fig S3); however, there was a statistical correlation between the latest versions of the two databases (Supplementary Fig. S2). In the future, the databases containing data on *Penicillium* are expected to improve. The relative abundance of *Penicillium* as determined via the culture-based method was consistently higher than those from the DNA-based method regardless of the databases used. The similar tendency, that the relative abundances of *Penicillium* by the culture-based method were consistently higher than by the DNA-based method, was also reported by previous studies^8,28^. We expect that the underestimation by the DNA-based method is partly attributable to possible primer bias associated with the fITS7 primer that was used in this study and is known to exclude certain *Penicillium* species^29^. The causes of this difference are an issue that requires further studies.

Yeasts such as *Sterigmatomyces* and *Blastobotrys* were detected using the DNA-based method (Fig. 3), but rarely from the culture-based method (Supplementary Fig. S1). One reason could be that some yeasts are dimorphic and adopt yeast or filamentous forms depending on culture conditions^21^. Some dimorphic species might not form yeast-like colonies on DG18 agar, which could be a reason of why the yeasts were underestimated via the culture-based method in this study. Additional media such as Dichloran Rose Bengal Chloramphenicol medium and Dixon’s medium are necessary for identification and determination of yeasts via culture techniques.

## Methods

### Dust sampling

As part of our previous sampling campaign^9,10^, house dust samples collected for 70–100 min in 24 unoccupied houses in Fukushima prefecture in Japan from April 2017 to May 2018—except for August 2017 and January through February 2018—, were analyzed in this study. House dust (20–63 μm) was sampled by particle size using a cyclone vacuum (DC61MH; Dyson) with 20-μm and 63-μm stainless sieves (φ75 × 20 mm, 5–3294-54 and 5-3294-46; SANPO Co., Saitama, Japan). Dust samples were additionally sieved in the laboratory, weighed with a balance, and then stored at 4 °C. Our dust sampling methods have been reported in greater detail by previous studies in which the radioactivity of house dust was determined^9,10^. After the sampling, although most dust samples were stored at 4 °C for 3 weeks to 1 month until cultivation; however, a few samples were stored at 4 °C for 3 months until cultivation since Toyozaki^20^ reported that spores can survive at 4 °C for several months. Then, dust samples were stored at -80 °C for 6–18 months until DNA sequencing.

### Culture-based method

Approximately 50 mg of house dust was weighed and then stirred for 1 min in 10 mL of a PBS solution containing 0.05% Tween20. The suspension (0.5 mL) was injected onto DG18 agar (Dichloran 18% glycerol agar) plates with water activity adjusted at 0.95. Fungi on these plates were cultured at 25 °C for 7–14 days for subsequent counting. If the number of colonies on a plate exceeded approximately 100–200, which could not be counted as the fungal colonies frequently overlapped, the suspension was diluted with PBS solution containing 0.05% Tween-20, reinjected onto a plate, cultured, and counted. For the species whith low relative abundances, the number of colonies were counted from a pre-diluted plate. Isolated fungi were identified according to their colony and microscopic characteristics after subculturing on potato dextrose agar (PDA), malt extract agar (MEA), and Czapek yeast extract agar (CYA) plates^21,30^.

### DNA extraction

DNA was extracted from approximately 10 mg of each house dust sample using a PowerMax Soil DNA Isolation Kit (Mobio Laboratory, Carlsbad, CA, USA). We followed the kit’s protocol with a modified step for sample homogenization with supplementary glass beads 0.1 mm and 0.5 mm in diameter (300 mg and 100 mg, respectively) for 3 min using a bead beater (BioSpec Products, Bartlesville, OK, USA)^31^. The DNA was purified and eluted with 50 μL of TE (10 mM Tris–HCl, 1 mM EDTA, pH 8.0).

### DNA sequencing

The fungal internal transcribed spacer 2 (ITS2) region was amplified with the fungal primers fITS7 (5′-GTGARTCATCGAATCTTTG-3′) and ITS4 (5′-TCCTCCGCTTATTGATATGC-3′) [28,32,] with the Illumina Miseq adapter sequences. PCR was conducted in a 30-μL reaction mixture comprising 0.33 μM of each primer, 2 × PCR Solution Premix Taq DNA polymerase (Takara Bio Inc., Otsu, Shiga, Japan), and 1 μL of DNA extract on a T100 thermal cycler (Bio-Rad Laboratories, Inc., Hercules, CA, USA). Thermal conditions were as reported elsewhere^33^. After PCR, AMPure XP beads (Beckman Coulter, Inc., Brea, CA, USA) were used to purify PCR amplicons. Using a Nextera XT Index kit (Illumina, Inc., San Diego, CA, USA), index PCR was performed in a 50-μL reaction mixture comprising 5 μL of each index primer, 2 × PCR Solution Premix Taq DNA polymerase (Takara Bio), and 5 μL of the purified DNA. The thermal conditions were 3 min at 95 °C, followed by 10 cycles of 30 s at 95 °C, 30 s at 55 °C, and 30 s at 72 °C. The final elongation step was performed for 5 min at 72 °C. After the index PCR, the indexed PCR amplicons were purified using AMPure XP beads. Each indexed and purified amplicon was normalized to 4 nM with 10 mM Tris–HCl (pH 8.5) and pooled with PhiX (30%). The pooled libraries were loaded onto a v3 600 cycle-kit reagent cartridge (Illumina) for 2 × 300 bp paired-end sequencing by Illumina MiSeq. Raw sequence data are available under the project number PRJNA605669 in the Sequence Read Archive (SRA) of the National Center for Biotechnology Information (NCBI).

### DNA sequence analyses

Sequence reads with quality scores below 20 were excluded using the MiSeq Reporter v2.5 software package (Illumina). Trimmomatic-0.38^34^ was used to remove ambiguous base calls, and QIIME v1.9.1^35^ was used to join forward and reverse sequence reads with a minimum allowed overlap of 10 bp. Chimeric reads were identified against the reference database uchime_reference_dataset_ITS2_28.06.2017.fasta^36^ and removed by the chimera.vsearch command using mothur v1.41.3^37^. On the Galaxy platform^38^, the resultant sequences were further filtered with a minimum threshold length of 100 bp. After quality trimming and filtering, the remaining sequences were taxonomically assigned by the BLASTN2.2.28 + program^39^ against the latest UNITE reference database version 8.2^40^, downloaded on October 07, 2020 and classified by FHiTINGS v1.4^41^. To check for potential database biases, the sequences were also searched against the fungal ITS RefSeq database (PRJNA177353)^42^, that was downloaded on October 07, 2020. For diversity analyses, the sequences were clustered into operational taxonomic units (OTUs) with a 97% sequence similarity threshold^43–45^. From each of 24 analyzed libraries, 10,000 sequences were subsampled for diversity analyses using mothur v1.41.3^37^. As DNA sequencing of ITS is insufficient to identify species in most genera^46^, species identification was not conducted in the present study.

### Statistical analysis

Statistical analyses were conducted using SPSS 20.0 (IMD SPSS, Armonk, NY, USA). The Kolmogorov–Smirnov test was conducted to assess whether the relative abundances of fungal genera or species were normally distributed. Where no departure from normality was detected, Pearson’s correlation analysis was used to test the correlation between the relative abundances of fungal genera obtained from the culture and sequencing-based methods. Spearman’s correlation was employed for non-normally distributed data. The paired t-test (for normally distributed samples) and the paired Wilcoxon signed-rank test (for non-normal samples) were also used to compare the relative abundance data obtained by the culture and sequencing methods.

## Supplementary Information

Supplementary Information.
