# Supplementary material for: Comparison of DNA sequencing and morphological identification techniques to characterize environmental fungal communities
Source: Sci Rep. 2021 Jan 29;11:2633. doi: 10.1038/s41598-021-81996-w (PMC7846767; doi:10.1038/s41598-021-81996-w)
Supplement: Supplementary file 1 — Supplementary Information. [file 41598_2021_81996_MOESM1_ESM.docx]

**SUPPLEMENTARY MATERIALS FOR**

**Comparison of DNA sequencing and morphological identification techniques to characterize environmental fungal communities**

Naohide Shinohara^a*^, Cheolwoon Woo^b^, Naomichi Yamamoto^b^, Kazuhiro Hashimoto^c^, Hiroko Yoshida-Ohuchi^d^, Yuji Kawakami^c^

^a^ Research Institute of Science for Safety and Sustainability (RISS), National Institute of Advanced Industrial Science and Technology (AIST), 16-1 Onogawa, Tsukuba, Ibaraki 305-8569, Japan

^b^ Department of Environmental Health Sciences, Graduate School of Public Health, Seoul National University, Seoul 08826, Republic of Korea

^c^ Laboratory of Integrated Pest Management, FCG Research Institute Inc., 1-1-20, Aomi, Koto-ku, Tokyo 135-0064, Japan

^d^ Graduate School of Pharmaceutical Sciences, Tohoku University, 6-3 Aramaki-Aoba, Aoba-ku, Sendai, Miyagi 980-8578, Japan

* *Correspondence to:* Naohide Shinohara; e-mail: [n-shinohara@aist.go.jp](mailto:n-shinohara@aist.go.jp); TEL: +81-29-861-8030, FAX: +81-29-861-8411

**Fig. S1 Relative abundances of fungal species based on the culture method.**

**Fig. S2. Correlation of relative abundances based on the UNITE and RefSeq databases.**

**Fig. S2. (cont.)**

**Fig. S2. (cont.)**

**Fig. S3. Correlation of the results from Culture based method and DNA-based method in the previous database (Left) and Correlation of relative abundances based on the UNITE and RefSeq databases in the previous database (Right).**

**Table S1. Most abundant fungal genera.** Fungal genera with the highest 12 and 40 relative abundance levels in house dust (20–63 μm), based on culture and sequencing methods, in 24 Japanese houses.
